# The First Report of a Fully Sequenced Resistance Plasmid from *Shigella boydii*

**DOI:** 10.3389/fmicb.2016.01579

**Published:** 2016-10-06

**Authors:** Li Wang, Lei Liu, Dong Liu, Zhe Yin, Jiao Feng, Defu Zhang, Haihong Fang, Yefeng Qiu, Weijun Chen, Ruisheng Yang, Jinglin Wang, Yunzhi Fa, Dongsheng Zhou

**Affiliations:** ^1^Department of Clinical Laboratory, the First Hospital Affiliated to Henan UniversityKaifeng, China; ^2^State Key Laboratory of Pathogen and Biosecurity, Beijing Institute of Microbiology and EpidemiologyBeijing, China; ^3^College of Food Science and Project Engineering, Bohai UniversityJinzhou, China; ^4^Laboratory Animal Center, Academy of Military Medical SciencesBeijing, China; ^5^Beijing Institute of Genomics (CAS)Beijing, China

**Keywords:** *Shigella boydii*, p2246-CTXM, *bla*_CTX-M-14_, *erm(B)*, *mph(A)*

## Abstract

The purpose of this study was to characterize mechanisms of plasmid-mediated antimicrobial resistance in *Shigella boydii*. *S. boydii* strain 2246 with resistance to ciprofloxacin, ceftriaxone and azithromycin was isolated from a human case of watery diarrhea in a Chinese public hospital. Resistance in strain 2246 to ceftriaxone and azithromycin was attributable to the presence of *bla*_CTX-M-14_, and *erm(B)* and *mph(A*), respectively, which were co-located on a multidrug-resistant (MDR) plasmid p2246-CTXM. p2246-CTXM represented a novel IncFII-type MDR plasmid with a very complex chimera structure. Its master backbone was genetically closely related to the R100 plasmid, but p2246-CTXM had evolved to integrate additional R100-unrelated backbone regions as well as massive exogenous mobile elements that carried multiple resistance determinants. In p2246-CTXM*, erm(B)* together with its leading peptide gene *erm(C)*, *mph(A*) together with its regulatory genes *mrx* and *mphR(A)*, and *bla*_CTX-M-14_ were captured by three different mobile elements Tn*6295*, the IS*26*-*mph(A)*-*mrx*-*mphR(A)*-IS*6100* unit, and a truncated IS*Ecp1*-*bla*_CTX-M-14_-IS*903D*-*iroN* transposition unit, respectively, all of which were harbored in a large Tn*3*-family transposon Tn*6285*. p2246-CTXM still carried additional resistance determinants *mer* (mercury resistance), *aacA4* (aminoglycoside resistance), *cmlA1* (chloramphenicol resistance), and *qacED1* (quaternary ammonium compound resistance). This is the first report of identifying a clinical *S. boydii* strain simultaneously resistant to ciprofloxacin, ceftriaxone, and azithromycin, and determining the complete sequence of a resistance plasmid from *S. boydii*.

## Introduction

Shigellosis remains a common gastrointestinal disease in both developing and industrialized countries. *Shigella*, the causative agent of shigellosis, can be serologically grouped into four species *S. flexneri*, *S. sonnei*, *S. boydii*, and *S. dysenteriae*. Being together responsible for about 90% of shigellosis, *S. flexneri* and *S. sonnei* are the most prevalent species in developing and developed countries, respectively, but a shift in the dominant species from *S. flexneri* to *S. sonnei* has occurred in countries with recent rapid improvement of socioeconomic conditions (Livio et al., 2014; Lima et al., 2015). *S. boydii* accounts for around 10% of the *Shigella* isolates found in the samples from the Indian subcontinent that is considered as *S. boydii* endemic areas, but this pathogen remains very rare (less than 1% of the total *Shigella* isolates) in other areas (Livio et al., 2014; Lima et al., 2015).

Multidrug-resistant (MDR) isolates of *S. flexneri* and *S. sonnei* have been reported worldwide. The previously efficacious old-generation antimicrobials such as ampicillin, chloramphenicol, tetracycline, and sulphonamides have become ineffective in the treatment of shigellosis (Niyogi, 2007; Zhang W. et al., 2011). The situation is getting worse due to the increasing emergence of resistance in *Shigella* to ciprofloxacin, ceftriaxone, and azithromycin (Klontz and Singh, 2015). The WHO recommends ciprofloxacin as the first choice for the treatment of multidrug-resistant shigellosis, while ceftriaxone and azithromycin can be used as the alternatives for both adults and children and they are preferred among young children because of the concerns regarding the adverse effects of ciprofloxacin in young children (Christopher et al., 2010).

This study describes not only the first reported *S. boydii* isolate with resistance to ciprofloxacin, ceftriaxone and azithromycin but also the first fully sequenced resistance plasmid from *S. boydii*. This plasmid, designated p2246-CTXM, is a novel MDR plasmid of the IncFII incompatibility group, and it carries several different resistance determinants especially including *bla*_CTX-M-14_ (cephalosporin resistance), and *erm(B)* and *mph(A*) (macrolide resistance).

## Materials and Methods

### Bacterial Identification

The use of human specimens and all related experimental protocols were approved by the Committee on Human Research of indicated institutions and carried out in accordance with the approved guidelines. *S. boydii* species was identified by slide agglutination using monovalent antisera of Denka Seiken (Tokyo, Japan) and monoclonal antibody reagents of Reagensia AB (Solna, Sweden). The agglutination was sensitively scored according to the following scale: +++, 100% agglutination of the cells; ++, >50% agglutination; +, <50% agglutination; -, no agglutination detected.

### Detection of Resistance Genes

The major horizontally acquired quinolone-resistance genes, extended-spectrum β-lactamase (ESBL) genes, and macrolide-resistance genes were screened by PCR (Supplementary Table S1), followed by amplicon sequencing on ABI 3730 Sequencer (LifeTechnologies, Carlsbad, CA, USA).

### Plasmid Transfer, Sequencing and Annotation

Plasmid conjugal transfer were performed with *Escherichia coli* EC600 (LacZ^-^, Nal^R^, and Rif^R^) being used as recipient for selection of *bla*_CTX-M_-positive transconjugants (Chen et al., 2015). Plasmid DNA was isolated from *E. coli* transconjugant using Qiagen large construct kit (Qiagen, Hilden, Germany), and sequenced by whole-genome shotgun strategy in combination with Illumina HiSeq 2500 (Illumina, San Diego, CA, USA) sequencing technology. The contigs were assembled with Velvet version 1.2, and the gaps were filled through combinatorial PCR and Sanger sequencing on ABI 3730 Sequencer. The genes were predicted with GeneMarkS^TM^ and RAST and further annotated by BLASTP and BLASTN against UniProt and NR databases. Annotation of mobile elements was based on the databases ISfinder, INTEGRALL, Tn Number Registry, and ISCR Elements. Gene organization diagrams were drawn with Inkscape version 0.48.

### Phenotypic Analyses

Enzymatic activity of ESBL was determined by the combined disk test as recommended by Clinical and Laboratory Standards Institute (CLSI; CLSI, 2015). Bacterial antimicrobial susceptibility was tested by ETEST (BioMérieux, Hazelwood, MO, USA) for erythromycin and azithromycin and by VITEK 2 (BioMérieux Vitek, Hazelwood, MO, USA) for all the other drugs (**Table 1**), and interpreted as per CLSI guidelines (CLSI, 2015).

**Table 1:** Antimicrobial drug susceptibility profiles.

Category	Antibiotics	MIC (mg/L)/antimicrobial susceptibility		
		2246	2246-CTXM -EC600	EC600
Penicillins	Piperacillin	≥128R	≥128R	≤4S
	Piperacillin/tazobactam	8S	≤ 4S	≤4S
Cephalosporins	Cefazolin	≥64R	≥64R	≤4S
	Cefuroxime	≥64R	≥64R	16I
	Ceftriaxone	≥64R	≥64R	≤1S
Macrolides	Erythromycin^a^	≥256	≥256	12
	Azithromycin^a^	≥256	≥256	2
Fluoroquinolones	Ciprofloxacin	≥4R	≤0.25S	≤0.25S
	Levofloxacin	≥8R	1S	0.5S
Carbapenems	Imipenem	≤1S	≤1S	≤1S
	Meropenem	≤0.25S	≤0.25S	≤0.25S
Aminoglycosides	Amikacin	≤2S	≤2S	≤2S
	Gentamicin	≤1S	≤1S	≤1S
Sulfanilamides	Trimethoprim/sulfamethoxazole	≤20S	≤20S	≤20S

*S = sensitive; R = resistant. ^a^’R’ and ’S’ cannot by judged due to because erythromycin an azithromycin susceptibility testing guidelines and interpretive criteria are currently lacking for Shigella.*

### RNA Isolation and Primer Extension Assay

Bacteria were cultured overnight in Mueller-Hinton broth with addition of indicated antibiotics. Total RNAs were extracted from harvested bacterial cells using TRIzol Reagent (Life Technologies). The oligonucleotide primers 5′-GCA CTCTCTTTGTCACCATCTC-3′, 5′-CTACATAACGCATTTGA TAACGC-3′, and 5′-CCATGTCGGGCTGCAAGTGCGTACAG TTGGG-3′ were designed to be complementary to the RNA transcripts of *bla*_CTX-M-14_, *erm(B)L* and *mph(A)*, respectively, and end-labeled with [γ-^32^P] ATP. About 10 μg of end-labeled primer were annealed with 3 μg of RNA for the primer extension assay with a Primer Extension System (Promega) as described previously (Zhang Y. et al., 2011). The end-labeled primer was used for sequencing the PCR amplicons generated by the primer pairs 5′-TTAAGTATCATTGCAGCAAAG-3′/5′-GCA CTCTCTTTGTCACCATCTC-3′, 5′-AACGCTGAACCCAGCG GTAAATCGT-3′/5′-TACTCCTGAAGTGATTACATCT-3′, and 5′-ATGGCAAACTGAAACGGAT-3′/5′-CCTCTGGTTCGACC TTCG-3′ for *bla*_CTX-M-14_, *erm(B)L*, and *mph(A)*, respectively. DNA sequencing was carried out using the AccuPower & Top DNA Sequencing Kit (Bioneer). The primer extension products and sequencing materials were analyzed on an 8 M urea-6% polyacrylamide gel electrophoresis. Radioactive species were detected by autoradiography.

### Nucleotide Sequence Accession Number

The complete sequence of p2246-CTXM was submitted to GenBank under accession number KX646543.

## Results

### Characterization of *S. boydii* Strain 2246 and Its Drug Susceptibility

In October 2013, a 51-year-old male with watery diarrhea and liver dysfunction (further diagnosed as liver cirrhosis) was admitted to a public hospital in Beijing, China. Bacterial growth could be observed after culture of fresh stool specimens of the patient on the Salmonella-Shigella agar. The bacterial isolate, designated as 2246, was identified as *S. boydii* using slide agglutination. Based on the antimicrobial susceptibility test results, the patient received oral administration with piperacillin/tazobactam, and his symptoms of diarrhea progressively disappeared.

As determined by PCR screening for the major plasmid-borne quinolone-resistance, ESBL, and macrolide-resistance genes, strain 2246 harbored *bla*_CTX-M-14_, *erm(B)*, and *mph(A*) rather than any of the other genes tested. These three resistance markers could be co-transferred from strain 2246 into EC600 through conjugation, generating a transconjugant 2246-CTXM-EC600. Strains 2246 and 2246-CTXM-EC600 had the ESBL enzyme activity (**Supplementary Figure S1**). Both 2246 and 2246-CTXM-EC600 were resistant to piperacillin, cefazolin, cefuroxime, and ceftriaxone, but remained susceptible to piperacillin/tazobactam, imipenem and meropenem, gentamicin and amikacin, and trimethoprim/sulfamethoxazole; 2246 rather than 2246-CTXM-EC600 was resistant to ciprofloxacin and levofloxacin (**Table 1**). Notably, both 2246 and 2246-CTXM-EC600 possessed greatly elevated minimal inhibitory concentration (MIC) values (≥256 mg/L) against erythromycin and azithromycin (**Table 1**).

### General Features of p2246-CTXM

**FIGURE 1:**
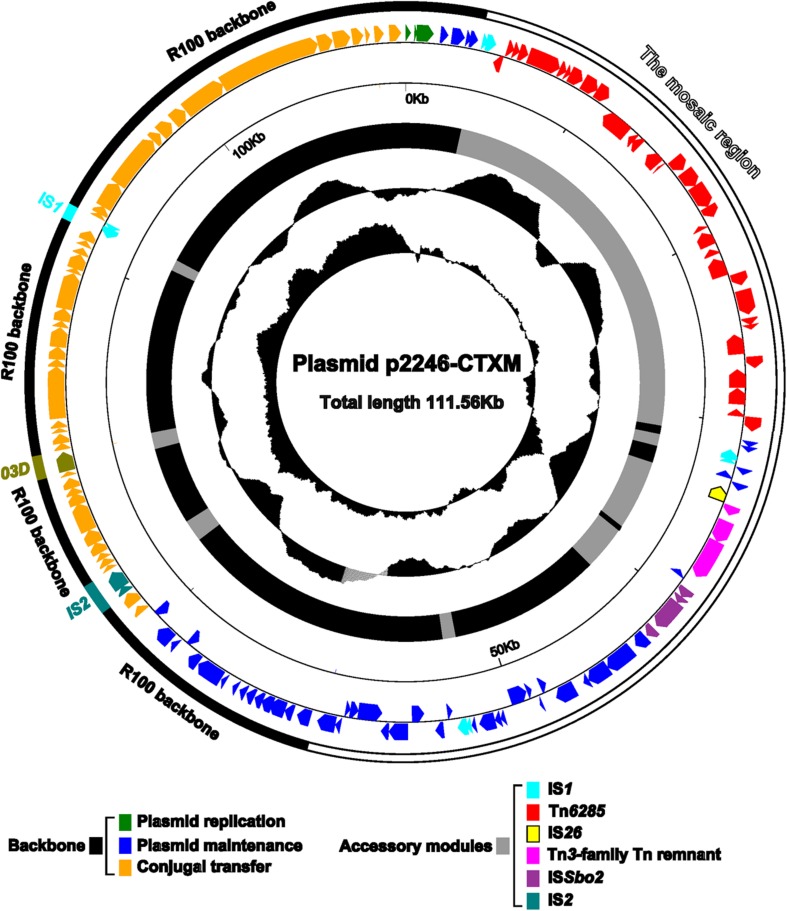
**Schematic map of p2246-CTXM.** Genes are denoted by arrows and colored based on gene function classification. The innermost two circles present the GC-Skew [(G-C)/(G+C)] and the GC contents. Shown also are the backbone and the accessory modules, respectively.

High-throughput sequencing analysis using plasmid DNA sample isolated from 2246-CTXM-EC600 generated the entire nucleotide sequence of a single plasmid p2246-CTXM (**Figure 1**) with a mean coverage of 135. The p2246-CTXM genome was manifested as an 111,559 bp circular DNA molecular with a mean G+C content of 51.9% and carried a total of 143 predicted open reading frames (ORFs).

The modular structure of p2246-CTXM could be divided into two major regions, namely, a 55-kb plasmid R100 (accession number AP000342)-derived backbone region with insertion of three different insertion sequences, and a 56.4-kb mosaic region composed of several plasmid backbone sequences and accessory mobile elements of different evolutionary origins. The R100-derived backbone sequences contained DNA regions for plasmid replication (*repA2*, *repA6*, and *repA1* of the IncFII incompatibility group), maintenance (*repA4*, *tir*, *pemIK*, etc) and conjugal transfer (*tra*, *trb*, *yfiABC*, etc). One copy of each of IS*2*, IS*903D* and IS*1* was inserted into the R100-derived backbone sequences, disrupting them into four separate portions. The transposition of IS*2*, IS*903D* and IS*1* leaved three different direct repeats (DRs: target site duplication signals of transposition), i.e., GTTTA, GCATAAATC, and CGCGACGGG, which flanked the corresponding inserted elements, respectively. The insertion of IS*2* and IS*903D* truncated the backbone genes *traJ* and *yfhA*, respectively.

Located at different sites of the backbone of R100 were three copies of repeat *D*, around 438 bp in length, two of which were found in p2246-CTXM. It seemed that the repeat *D*-mediated homologous recombination led to the deletion of one copy of repeat *D* together with a 14-kb region containing plasmid maintenance genes *ssb*, *ydeAB*, *ydgA*, *yefA*, *psiBA*, *sok*, *mok, hok*, and *yehA*.

### The Mosaic Region of p2246-CTXM

**FIGURE 2:**
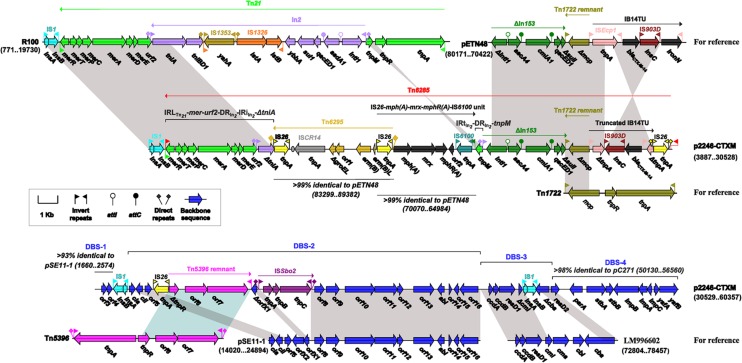
**The mosaic region of p2246-CTXM with comparison to similar elements.** Genes are denoted by arrows and colored based on gene function classification. Shading regions denote shared DNA regions of homology (>95% nucleotide similarity).

The 56.4-kb mosaic region of p2246-CTXM (**Figure 2**) could be divided into two parts and the first part was composed of an intact IS*1* element, and a novel transposon designated Tn*6285* bordered by 40 bp imperfect terminal inverted repeats (IRs) at both ends.

Tn*21*, as observed in plasmid R100, was a Tn*3*-family unit transposon which was sequentially organized as the core transposition module *tnpA* (transposase)-*tnpR* (resolvase), *tnpM*, the Tn*402*-related class 1 integron In*2* that was delimited by 25 bp IRs [inverted repeat initial (IRi) plus inverted repeat terminal (IRi)] associated 5 bp DRs, *urf2*, and the *mer* gene cluster (Liebert et al., 1999).Tn*21* was bordered by 38 bp IRs [inverted repeat left (IRL) plus inverted repeat right (IRR)] at both ends. It was though that the insertion of In*2* into Tn*21* disrupted a presumed ancestral *urf2M* gene into two separate ones *urf2* and *tnpM*.

A Tn*21* remnant, which comprised two separate fractions IRL_Tn_*_21_*-*mer*-*urf2*-DR_In_*_2_*- IRi_In_*_2_*-*ΔtniA* and IRt_In_*_2_*-DR_In_*_2_*-*tnpM*, was found in Tn*6285* (**Figure 2**). Separation of these two fraction was likely resulted from the insertion of two consecutive resistance elements Tn*6295* (name given in this study) and IS*26*-*mph(A)*-*mrx*-*mphR(A)*-IS*6100*, which were found at two different locations in pETN48 (accession number FQ482074), a CTX-M-14-encoding plasmid of the FII-FIB replicon type and originating from an *E. coli* O102-ST405 strain (Billard-Pomares et al., 2011).

Tn*6295* (**Figure 2**) was a composite transposon, which had two flanking copies of IS*26* oriented in the same direction and was further delimited by DRs of GGA at both ends; moreover, an IS*CR14* element and a macrolide-resistance operon *erm(B)L* (leading peptide)-*erm(B)* (methylase) were found in Tn*6295*.The IS*26*-*mph(A)*-*mrx*- *mphR(A)*-IS*6100* unit was known as a mobile element, which harbored another macrolide-resistance operon *mph(A)*-*mrx*-*mphR(A)* encoding a phosphotransferase, a positive regulator and a negative transcription factor, respectively (Partridge, 2011). Notably, the upstream IS*26* copy of Tn62*95* and the counterpart of the IS*26*-*mph*-IS*6100* unit overlapped each other in Tn*6285*, indicating that the connection of Tn62*95* with IS*26*-*mph(A)*-*mrx*-*mphR(A)*-IS*6100* was mediated by homologous recombination in *IS26*.

Upstream of *tnpM* in Tn*6285* was a 9.5 kb region, which was composed in order of ΔIn153, a Tn1*722* remnant, a truncated version of the IS*Ecp1*-*bla*_CTX-M-14_-based transposition unit (IB14TU), and IS*26* (**Figure 2**). This region was highly similar to that from nucleotide position 80171–70422 of pETN48. The *tnpM* gene was flanked by IRt_In_*_2_* and IRi_In_*_153_*, and it appeared that the homologous recombination mediated by these two 25 bp IRs characteristic of class 1 integrons led to the inversion of *tnpM* in Tn*6285* relative to Tn*21*. The ΔIn*153* in Tn*6285* contained two resistance gene cassettes *aacA4:attC_aacA4_* and *cmlA1:attC_cmlA1_* and this class 1 integron had undergone the truncation of *sulI* and the loss of IRt in the 3′-conserved segment. Tn*1722* was a Tn*3*-family cryptic transposon initially identified in *E. coli*, with a modular structure IRL_Tn_*_1722_*-*tnpA*-*tnpR*-*mcp* (methyl-accepting chemotaxis protein)-IRR_Tn_*_1722_* (Allmeier et al., 1992). The Tn1*722* remnant in Tn*6285* manifested as *Δmcp*-IRR_Tn_*_1722_*.

pETN48 carried a prototype IB14TU, bound by 14 bp terminal IRs, which contained *bla*_CTX-M-14_ together with an upstream IS*Ecp1* and a downstream IS*903D* followed by *ΔiroN.* Similar structures of IRL_IS_*_Ecp1_*-*tnpA*_IS_*_Ecp1_*-IRR_IS_*_Ecp1_*-*bla*_CTX-M_-IS*903D*-*ΔiroN*- IRR_IS_*_Ecp1_* were found in various plasmids of global origin (Zong et al., 2011). Compared with pETN48, Tn*6285* harbored a truncated IB14TU element, which had undergone the loss of *tnpA*_574to973_ (position 574 to 973 of the total 1263 bp coding region of *tnpA* in IS*Ecp1*) and *ΔiroN*-IRR_IS_*_Ecp1_*, and the inversion of *tnpA*_974to1263_-IRR_IS_*_Ecp1_*-*bla*_CTX-M-14_-IS*903D* (**Figure 2**).

The second part of the 56.4-kb mosaic region contained four plasmid backbone-derived sequences, designated DBS-1to DBS-4 in the order that they occur (**Figure 2**). DBS-1 was a 926 bp region with two annotated genes encoding hypothetical proteins, which showed 93% sequence similarity to the IncFII-type plasmid pSE11-1 from *E. coli* (Oshima et al., 2008). Located between DBS-1 and DBS-2 was an IS*1* element. The 18.7-kb DBS-2 contained the colicin Ia activity-immunity system *cia*–*cii*, and it was highly similar to a 15-gene region of pSE11-1. Two separate insertions, i.e., IS*26* connected with a Tn*5396* remnant, and a novel insertion sequence designated IS*Sbo2*, occurred within DBS-2 relative to pSE11-1. Tn*5396* was a Tn*3*-family cryptic transposon initially identified in the small plasmid pEC22 from *E. coli*, with a modular structure IRL_Tn_*_5396_*-*tnpA*-*tnpR*-*orf6 -orf7*-IRR_Tn_*_5396_* (Elhai et al., 1994), and the 3′-end region *ΔtnpR*-*orf6-orf7*-IRR_Tn_*_5396_* constituted the Tn*5396* remnant observed in p2246-CTXM. The 3.6-kb DBS-3 was highly similar to a 7-gene region of the *E. coli* genome assembly FHI74 (accession number LM996602), which contained the toxin-antitoxin system *ccdBA*, the colicin M immunity-activity system *cmi*–*cma* and the colicin B system *cbi*–*cba*. An IS*1* element was inserted into DBS-3, leaving *cmi* and *cba* truncated. The 6.4-kb DBS-4 was found in the IncI1-type plasmid pC271 (accession number LN735561) from *E. coli* and contained several plasmid maintenance genes such as *parA*, *stbAB*, and *impCAB*.

### Expression of *bla_CTX-M-14_*, *erm(B)*, and *mph(A)*

**FIGURE 3:**
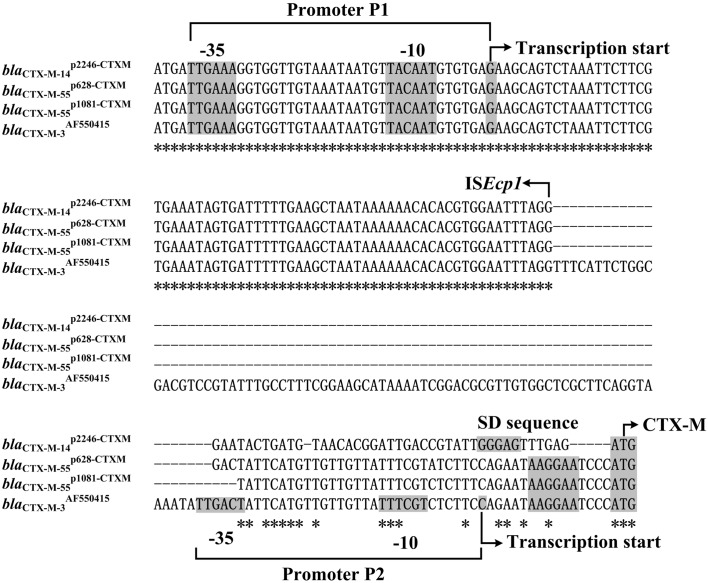
**Alignment of *bla*_CTX-M_ upstream sequences.** The 150- to 235-bp upstream sequences together with the start codon of the *bla*_CTX-M_ genes were aligned by MAFFT (http://www.ebi.ac.uk/Tools/msa/mafft/). Shown were core promoter regions, -35 and -10 elements, transcription starts, Shine–Dalgarno (SD) sequences for ribosome recognition, and translation starts.

Spacer regions between IS*Ecp1* and *bla*_CTX-M-55_ from different IS*Ecp1*-*bla*_CTX-M-55_ isoforms displayed three different lengths, namely 45 bp (e.g., *bla*_CTX-M-55_^p1081-CTXM^) (Qu et al., 2014), 48 bp (e.g., *bla*_CTX-M-55_^p2246-CTXM^ in this study), and 127 bp (e.g., *bla*_CTX-M-55_^JQ343851^ and *bla*_CTX-M-3_^AF550415^; **Figure 3**). A total of two promoters P1 and P2 were experimentally identified for *bla*_CTX-M-3_^AF550415^ with a 127 bp spacer and, moreover, the IS*Ecp1*-provided promoter P1 was stronger and more important than the intrinsic P2 promoter in the 127 bp spacer (Ma et al., 2011).

**FIGURE 4:**
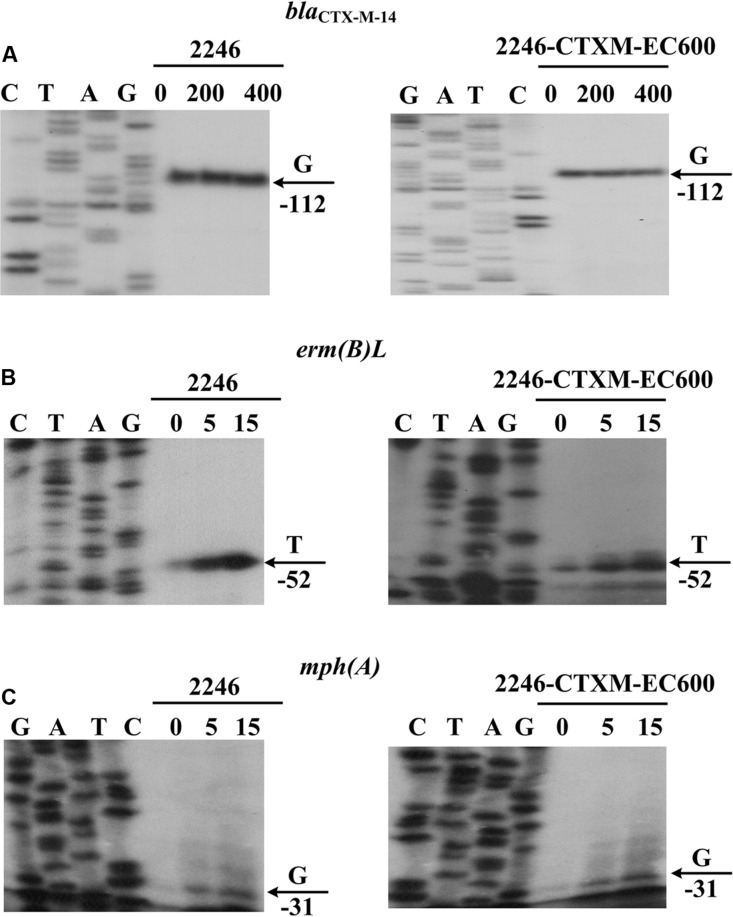
**Expression of resistance genes.** Primer extension assay of the RNA transcript of each of the genes *bla*_CTX-M-14_ **(A)**, *erm(B)L* **(B)** and *mph(A)* **(C)**, was performed for the two strains 2246 and 2246-CTXM-EC600 cultured with addition of increasing amounts of indicated antibiotics. Lanes C, T, A and G represent Sanger sequencing reactions. Lanes 0, 200, and 400 stand for 0, 200, and 400 mg/L ampicillin **(A)**, respectively. Lanes 0, 5, and 15 represent 0, 5, and 15 mg/L amikacin **(B)** or azithromycin **(C)**, respectively. Transcription start of each gene is indicated by arrow with nucleotide, and minus number under arrow indicates nucleotide position upstream of each gene. Representative data from at least two independent biological replicates are shown.

Compared with the 127 bp spacer, the 45 or 48 bp spacer from *bla*_CTX-M-55_^p1081-CTXM^ or *bla*_CTX-M-55_^p2246-CTXM^ was a truncated form due to the loss of an 82 or 79 bp region within the spacer, respectively (**Figure 3**). This deletion impaired the -35 element of P2, most likely making the P2 activity undetectable for *bla*_CTX-M-55_^p1081-CTXM^ and *bla*_CTX-M-55_^p2246-CTXM^. In the present study, the primer extension assay detected a single transcription start, i.e., nucleotide G located at 116 bp upstream of *bla*_CTX-M-55_ (**Figure 4**), which corresponded to the P1 promoter shared by *bla*_CTX-M-55_^p1081-CTXM^ (Qu et al., 2014) and *bla*_CTX-M-55_^p2246-CTXM^ (**Figure 3**). In addition, the primer extension assay showed that the addition of increasing amounts of ampicillin during cultivation of strains 1081 and 1081-CTXM EC600 had no effect on the activity of *bla*_CTX-M-55_^p2246-CTXM^ promoter (**Figure 4**), denoting the constitutive expression of *bla*_CTX-M-55_^p2246-CTXM^.

The primer extension assay detected a transcription start for each of *erm(B)L* and *mph(A)* from p2246-CTXM, i.e., nucleotide T located at 52 bp upstream of *erm(B)L*, and G at 31 bp upstream of *mph(A)*; in addition, this assay also showed the inducible expression of *erm(B)L* and *mph(A)* upon addition of increasing amounts of amikacin and azithromycin, respectively (**Figure 4**). The above results were consistent with the expression patterns previously characterized for the two macrolide-resistant operons *erm(B)L*-*erm(B)* (Min et al., 2008) and *mph(A)*-*mrx*-*mphR(A)* (Noguchi et al., 2000).

## Discussion

The current emergence and spread of resistance in *Shigella* strains to ciprofloxacin, ceftriaxone and azithromycin hinder the empirical antimicrobial therapy. Antimicrobial resistance patterns and mechanisms are overwhelmingly documented for *S. flexneri* and *S. sonnei*, and very little is currently known about *S. boydii* due to small number of available strains. This work describes a clinical *S. boydii* strain 2246 simultaneously resistant to ciprofloxacin, ceftriaxone, and azithromycin. As shown by PCR detection, strain 2246 contains none of the known horizontally acquired quinolone-resistance genes (data not shown). It seems that strain 2246 employs chromosomal-borne intrinsic mechanism(s) of quinolone resistance. Resistance in strain 2246 to ceftriaxone and azithromycin is attributable to the presence of *bla*_CTX-M-14_, and *erm(B)* and *mph(A*), respectively, which are co-located on a MDR plasmid p2246-CTXM.

CTX-M-type ESBLs have the high hydrolytic activity against cefotaxime and they can be divided now into five major phylogenetic groups, i.e, the CTX-M-1 group, the CTX-M-2 group, the CTX-M-8 group, the CTX-M-9 group, and the CTX-M-25 group (D’Andrea et al., 2013). CTX-M enzymes have showed the rapid increasing circulation in *Shigella* species in China during the past decade, and CTX-M-14 (a member of CTX-M-9 group), and CTX-M-15 and CTX-M-55 (belonging to the CTX-M-1 group) are listed sequentially as the top three common types of ESBL in the cefotaxime-resistant *S. flexneri* or *S. sonnei* from China (Zhang W. et al., 2011; Li et al., 2015).

Acquired resistance to macrolides in bacteria mainly result from three distinct mechanisms of resistance (Phuc Nguyen et al., 2009): target ribosome site modification by methylases [*erm(A)*, *erm(B)*, and *erm(C)*]; macrolide modification by esterases [*ere(A)* or *ere(B)*] or by phosphotransferases [*mph(A)*, *mph(B)*, and *mph(D)*]; and presence of efflux pumps [*mef(A)* and *msr(A)*]. Identification of azithromycin-resistant *Shigella* strains is rare and has been documented for only *S. flexneri* or *S. sonnei* from France (Boumghar-Bourtchai et al., 2008), the United States (Howie et al., 2010; Sjolund Karlsson et al., 2013) and Canada (Gaudreau et al., 2014), and all these azithromycin-non-susceptible *Shigella* isolates harbor the *mphA* gene. Notably, the p2246-CTXM plasmid coharbors *erm(B)* and *mph(A*), which account for two different transferable mechanisms of macrolide resistance.

Up to now, a total of seven virulence or cryptic *S. boydii* plasmids pBS512_211, pSB4_227, pBS512_2, pBS512_33, pBS512_5, pBS512_7, and pSB13 have their complete nucleotide sequences that ranged from 2 to 211 kb in size. This work presents the first fully sequenced resistance plasmid from *S. boydii*. p2246-CTXM represents a novel IncFII-type MDR plasmid with a very complex chimera structure. Its master backbone is genetically closely related to the R100 plasmid, but p2246-CTXM has evolved to integrate additional R100-unrelated backbone regions as well as massive exogenous mobile elements that carry multiple resistance determinants.

In p2246-CTXM*, erm(B)* together with its leading peptide gene *erm(C)*, *mph(A*) together with its regulatory genes *mrx* and *mphR(A)*, and *bla*_CTX-M-14_ are captured by three different transposable units Tn*6295*, IS*26*-*mph(A)*-*mrx*-*mphR(A)*-IS*6100*, and IB14TU, respectively, all of which are harbored in a large transposon Tn*6285*. p2246-CTXM still carries several additional resistance determinants *mer* (mercury resistance), *aacA4* (aminoglycoside resistance), *cmlA1* (chloramphenicol resistance), and *qacED1* (quaternary ammonium compound resistance). The coexistence of multiple resistance genes on the same transmissible plasmid might contribute to bacterial dissemination and persistence in various hosts and environments with different antimicrobial selections. Future surveillance and epidemiological studies are needed to evaluate the prevalence of p2246-CTXM-like plasmids among *Shigella* isolates in China and other countries.

## Author Contributions

DsZ, and YF designed experiments. LW, LL, DL, and ZY performed experiments. DsZ, and LW analyzed data. JF, DfZ, HF, YQ, WC, RY, and JW contributed reagents, materials and analysis tools. DsZ, YF, and LW, wrote this manuscript.

## Conflict of Interest Statement

The authors declare that the research was conducted in the absence of any commercial or financial relationships that could be construed as a potential conflict of interest.

## Supplementary Material

FIGURE S1 **The combined disk test.** Experiments are conducted with three plates inoculated with three independent bacterial cultures for each strain, and only a representative figure is shown herein. CTX: 30 μg of cefotaxime; CAZ: 30 μg of ceftazidime; CTX+CA: 30 μg of cefotaxime plus 10 μg of clavulanic acid; CAZ+CA: 30 μg of ceftazidime plus 10 μg of clavulanic acid. Clavulanic acid is a member of the β-lactam family of antibiotics with little or no intrinsic antibacterial activity of its own; instead, it is used to enhance the activity of antibiotics by blocking bacterial β-lactamases as a competitive inhibitor of β-lactamases. *A* ≥ 5 mm increased in the zone diameter for either cefotaxime (30 μg) or ceftazidime (30 μg) tested in combination with clavulanic acid (10 μg) versus its zone when tested alone indicates the ESBL activity in the indicated strain. The results showed the ESBL activity in strains 2246 and 2246-CTXM-EC600.
