# Supplementary figures and images for: The First Report of a Fully Sequenced Resistance Plasmid from Shigella boydii
Source: Front Microbiol. 2016 Oct 6;7:1579. doi: 10.3389/fmicb.2016.01579 (PMC5052281; doi:10.3389/fmicb.2016.01579)

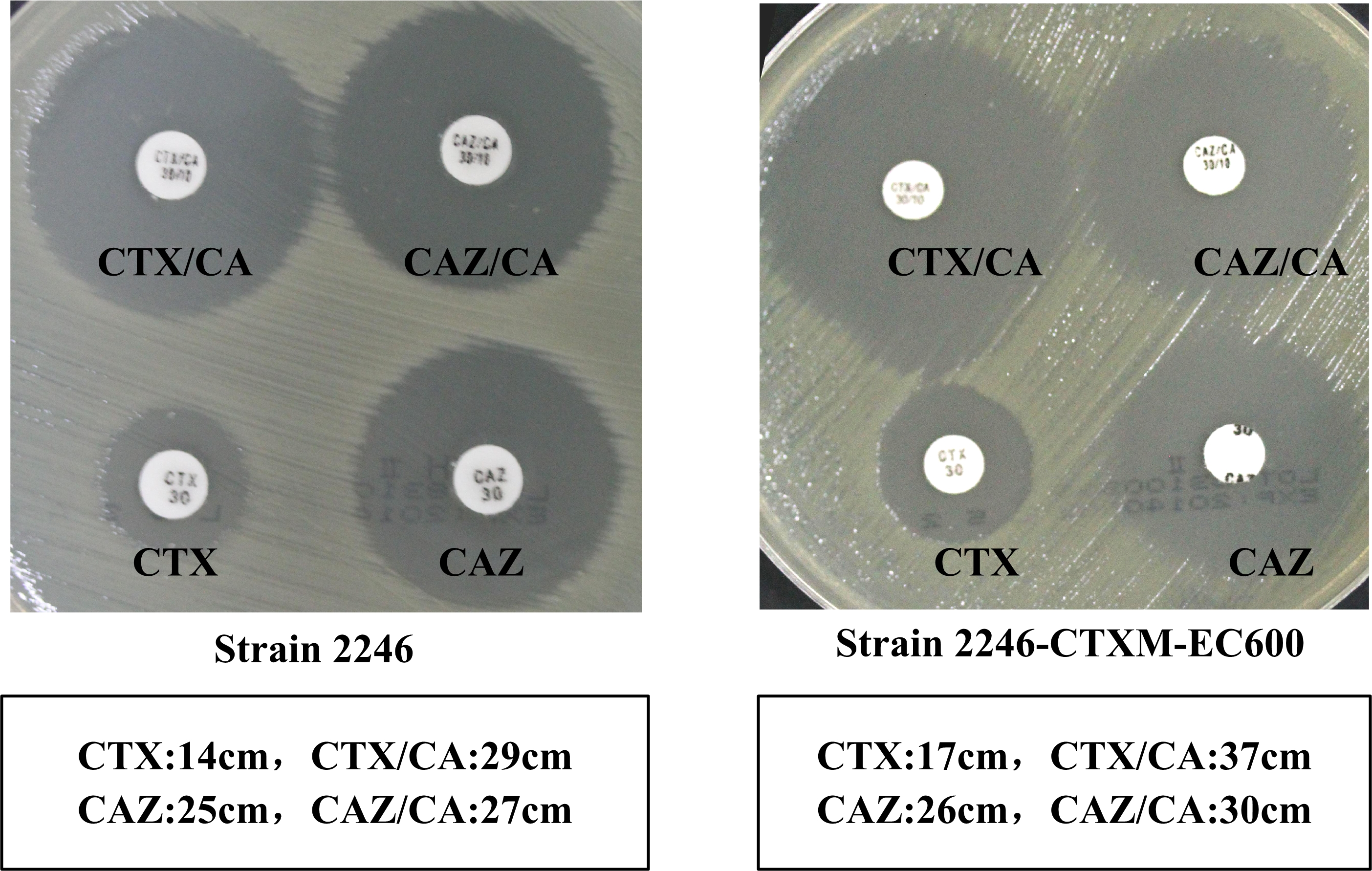

Supplement: FIGURE S1 — The combined disk test. Experiments are conducted with three plates inoculated with three independent bacterial cultures for each strain, and only a representative figure is shown herein. CTX: 30 μg of cefotaxime; CAZ: 30 μg of ceftazidime; CTX+CA: 30 μg of cefotaxime plus 10 μg of clavulanic acid; CAZ+CA: 30 μg of ceftazidime plus 10 μg of clavulanic acid. Clavulanic acid is a member of the β-lactam family of antibiotics with little or no intrinsic antibacterial activity of its own; instead, it is used to enhance the activity of antibiotics by blocking bacterial β-lactamases as a competitive inhibitor of β-lactamases. A ≥ 5 mm increased in the zone diameter for either cefotaxime (30 μg) or ceftazidime (30 μg) tested in combination with clavulanic acid (10 μg) versus its zone when tested alone indicates the ESBL activity in the indicated strain. The results showed the ESBL activity in strains 2246 and 2246-CTXM-EC600. [file Image_1.JPEG]
