# Supplementary material for: The First Report of a Fully Sequenced Resistance Plasmid from Shigella boydii
Source: Front Microbiol. 2016 Oct 6;7:1579. doi: 10.3389/fmicb.2016.01579 (PMC5052281; doi:10.3389/fmicb.2016.01579)
Supplement: Supplementary file 2 [file Table_1.DOCX]

**Table S1. PCR detection of horizontally acquired resistance genes**

| **Target gene** | **Primers** | **S-A** | **A-T** | **Refrence** |
| --- | --- | --- | --- | --- |
| **Extended-spectrum β-lactamases (ESBLs)** | | | | |
| *bla*_CTX-M_ universal | CTX-M-UF: ATGTGCAGYACCAGTAARGT  CTX-M-UR: TGGGTRAARTARGTSACCAGA | 593 | 52 | ([Pagani et al., 2003](#_ENREF_4)) |
| *bla*_CTX-M-1_ group | CTX-M-1GF: AAAAATCACTGCGCCAGTTC  CTX-M-1GR: AGCTTATTCATCGCCACGTT | 415 | 52 | ([Woodford et al., 2006](#_ENREF_9)) |
| *bla*_CTX-M-2_ group | CTX-M-2GF: CGACGCTACCCCTGCTATT  CTX-M-2GR: CCAGCGTCAGATTTTTCAGG | 552 | 52 | ([Woodford et al., 2006](#_ENREF_9)) |
| *bla*_CTX-M-8_ group | CTX-M-8GF: TCGCGTTAAGCGGATGATGC  CTX-M-8GR: AACCCACGATGTGGGTAGC | 666 | 52 | ([Woodford et al., 2006](#_ENREF_9)) |
| *bla*_CTX-M-9_ group | CTX-M-9GF2: ATGGTGACAAAGAGAGTGCA  CTX-M-9GR2: CCCTTCGGCGATGATTCTC | 869 | 52 | ([Barguigua et al., 2011](#_ENREF_1)) |
| *bla*_CTX-M-25_ group | CTX-M-25GF: GCACGATGACATTCGGG  CTX-M-25GR: AACCCACGATGTGGGTAGC | 327 | 52 | ([Woodford et al., 2006](#_ENREF_9)) |
| *bla*_TEM_ | TEM-F: CATTTCCGTGTCGCCCTTATTC  TEM-R: CGTTCATCCATAGTTGCCTGAC | 800 | 52 | ([Dallenne et al., 2010](#_ENREF_3)) |
| *bla*_SHV_ | SHV-F: AGCCGCTTGAGCAAATTAAAC  SHV-R: ATCCCGCAGATAAATCACCAC | 713 | 52 | ([Dallenne et al., 2010](#_ENREF_3)) |
| *bla*_GES_ | GES-ESBL-F: AGTCGGCTAGACCGGAAAG  GES-ESBL-R: TTTGTCCGTGCTCAGGAT | 399 | 52 | ([Dallenne et al., 2010](#_ENREF_3)) |
| *bla*_PER_ | PER-F: GCTCCGATAATGAAAGCGT  PER-R: TTCGGCTTGACTCGGCTGA | 520 | 52 | ([Dallenne et al., 2010](#_ENREF_3)) |
| *bla*_VEB_ | VEB-F: CATTTCCCGATGCAAAGCGT VEB-R: CGAAGTTTCTTTGGACTCTG | 648 | 52 | ([Dallenne et al., 2010](#_ENREF_3)) |
| *bla*_OXA-1_ group | OXA-1-F: GGCACCAGATTCAACTTTCAAG  OXA-1-R: GACCCCAAGTTTCCTGTAAGTG | 564 | 52 | ([Dallenne et al., 2010](#_ENREF_3)) |
| *bla*_OXA-2_ group | OXA-2-F: GACCAAGATTTGCGATCAGCAATGCG  OXA-2-R: CYTTGACCAAGCGCTGATGTTCYACC | 256 | 52 | ([Dallenne et al., 2010](#_ENREF_3)) |
| *bla*_OXA-10_ group | OXA-10-F: CGCCAGAGAAGTTGGCGAAGTAAG  OXA-10-R: GAAACTCCACTTGATTAACTGCGG | 138 | 52 | ([Dallenne et al., 2010](#_ENREF_3)) |
| **Quinolone resistance** | | | | |
| *qnrA* | qnrA-F1: CAGCAAGAGGATTTCTCACG  qnrA-R1: AATCCGGCAGCACTATTACTC | 630 | 56 | ([Ciesielczuk et al., 2013](#_ENREF_2)) |
| *qnrB* | qnrB-F1: GGCTGTCAGTTCTATGATCG  qnrB-R1: SAKCAACGATGCCTGGTAG | 488 | 56 | ([Ciesielczuk et al., 2013](#_ENREF_2)) |
| *qnrC* | qnrC-F1: GCAGAATTCAGGGGTGTGAT  qnrC-R1: AACTGCTCCAAAAGCTGCTC | 118 | 56 | ([Ciesielczuk et al., 2013](#_ENREF_2)) |
| *qnrD* | qnrD-F1: CGAGATCAATTTACGGGGAATA  qnrD-R1: AACAAGCTGAAGCGCCTG | 581 | 56 | ([Ciesielczuk et al., 2013](#_ENREF_2)) |
| *qnrS* | qnrS-F1: GCAAGTTCATTGAACAGGGT  qnrS-R1: TCTAAACCGTCGAGTTCGGCG | 428 | 56 | ([Ciesielczuk et al., 2013](#_ENREF_2)) |
| *qnrVC* | qnrVC-F1: GGATAAAACAGACCAGTTATATGTACAAG  qnrVC-R1: AGATTTGCGCCAATCCATCTATT | 444 | 56 | ([Tacao et al., 2014](#_ENREF_8)) |
| *aacA4cr* | aacA4cr-F1: TTGGAAGCGGGGACGGAM  aacA4cr-R1: ACACGGCTGGACCATA | 260 | 56 | ([Ciesielczuk et al., 2013](#_ENREF_2)) |
| *oqxAB* | oqxAB-F1: CCGCACCGATAAATTAGTCC  oqxAB-R1: GGCGAGGTTTTGATAGTGGA | 313 | 56 | ([Ciesielczuk et al., 2013](#_ENREF_2)) |
| *qepA* | qepA-F1: GCAGGTCCAGCAGCGGGTAG  qepA-R1: CTTCCTGCCCGAGTATCGTG | 218 | 56 | ([Ciesielczuk et al., 2013](#_ENREF_2)) |
| **Macrolide resistance** | | | | |
| *mph(A)* | mph(A)-F1: GTGAGGAGGAGCTTCGCGAG  mph(A)-R1: TGCCGCAGGACTCGGAGGTC | 403 | 56 | ([Phuc Nguyen et al., 2009](#_ENREF_5)) |
| *mph(B)* | mph(B)-F1: GATATTAAACAAGTAATCAGAATAG  mph(B)-R1: GCTCTTACTGCATCCATACG | 494 | 56 | ([Phuc Nguyen et al., 2009](#_ENREF_5)) |
| *mph(D)* | mph(D)-F1: AGCCAATTGCTACATGCGCTCT  mph(D)-R1: GGGTTTACGAGCCAAGCAAGAA | 756 | 56 | ([Soge et al., 2006](#_ENREF_7)) |
| *mph(E)* | mph(E)-F1: ATGCCCAGCATATAAATCGC  mph(E)- R1: ATATGGACAAAGATAGCCCG | 271 | 56 | ([Rose et al., 2012](#_ENREF_6)) |
| *erm(A)* | erm(A)-F1: TCTAAAAAGCATGTAAAAGAAA  erm(A)-R1: CGATACTTTTTGTAGTCCTTC | 533 | 56 | ([Phuc Nguyen et al., 2009](#_ENREF_5)) |
| *erm(B)* | erm(B)-F1: GAAAAAGTACTCAACCAAATA  erm(B)-R1: AATTTAAGTACCGTTACT | 639 | 45 | ([Phuc Nguyen et al., 2009](#_ENREF_5)) |
| *erm(C)* | erm(C)-F1: TCAAAACATAATATAGATAAA  erm(C)-R1: GCTAATATTGTTTAAATCGTCAAT | 642 | 45 | ([Phuc Nguyen et al., 2009](#_ENREF_5)) |
| *ere(A)* | ere(A)-F1: GCCGGTGCTCATGAACTTGAG  ere(A)-R1: CGACTCTATTCGATCAGAGGC | 420 | 56 | ([Phuc Nguyen et al., 2009](#_ENREF_5)) |
| *ere(B)* | ere(B)-F1: TTGGAGATACCCAGATTGTAG  ere(B)-R1: GAGCCATAGCTTCAACGC | 537 | 56 | ([Phuc Nguyen et al., 2009](#_ENREF_5)) |
| *mef(A)* | mef(A)-F1: AGTATCATTAATCACTAGTGC  mef(A)-R1: TTCTTCTGGTACTAAAAGTGG | 345 | 56 | ([Phuc Nguyen et al., 2009](#_ENREF_5)) |
| *msr(A)* | msr(A)-F1: GCACTTATTGGGGGTAATGG  msr(A)-R1: GTCTATAAGTGCTCTATCGTG | 384 | 56 | ([Phuc Nguyen et al., 2009](#_ENREF_5)) |
| *msr(E)* | msr(E)-F1: TATAGCGACTTTAGCGCCAA  msr(E)-R1: GCCGTAGAATATGAGCTGAT | 395 | 56 | ([Rose et al., 2012](#_ENREF_6)) |

S-A: size of amplicon (bp); A-T: Annelling temperature (ºC).

**References**

Barguigua, A., El Otmani, F., Talmi, M., Bourjilat, F., Haouzane, F., Zerouali, K., and Timinouni, M. (2011). Characterization of extended-spectrum beta-lactamase-producing Escherichia coli and Klebsiella pneumoniae isolates from the community in Morocco. *J Med Microbiol* 60**,** 1344-1352. doi: jmm.0.032482-0 [pii]

10.1099/jmm.0.032482-0.

Ciesielczuk, H., Hornsey, M., Choi, V., Woodford, N., and Wareham, D.W. (2013). Development and evaluation of a multiplex PCR for eight plasmid-mediated quinolone-resistance determinants. *J Med Microbiol* 62**,** 1823-1827. doi: 10.1099/jmm.0.064428-0.

Dallenne, C., Da Costa, A., Decre, D., Favier, C., and Arlet, G. (2010). Development of a set of multiplex PCR assays for the detection of genes encoding important beta-lactamases in Enterobacteriaceae. *J Antimicrob Chemother* 65**,** 490-495. doi: 10.1093/jac/dkp498.

Pagani, L., Dell'amico, E., Migliavacca, R., D'andrea, M.M., Giacobone, E., Amicosante, G., Romero, E., and Rossolini, G.M. (2003). Multiple CTX-M-type extended-spectrum beta-lactamases in nosocomial isolates of Enterobacteriaceae from a hospital in northern Italy. *J Clin Microbiol* 41**,** 4264-4269.

Phuc Nguyen, M.C., Woerther, P.L., Bouvet, M., Andremont, A., Leclercq, R., and Canu, A. (2009). Escherichia coli as reservoir for macrolide resistance genes. *Emerg Infect Dis* 15**,** 1648-1650. doi: 10.3201/eid1510.090696.

Rose, S., Desmolaize, B., Jaju, P., Wilhelm, C., Warrass, R., and Douthwaite, S. (2012). Multiplex PCR to identify macrolide resistance determinants in Mannheimia haemolytica and Pasteurella multocida. *Antimicrob Agents Chemother* 56**,** 3664-3669. doi: 10.1128/AAC.00266-12.

Soge, O.O., Adeniyi, B.A., and Roberts, M.C. (2006). New antibiotic resistance genes associated with CTX-M plasmids from uropathogenic Nigerian Klebsiella pneumoniae. *J Antimicrob Chemother* 58**,** 1048-1053.

Tacao, M., Moura, A., Correia, A., and Henriques, I. (2014). Co-resistance to different classes of antibiotics among ESBL-producers from aquatic systems. *Water Res* 48**,** 100-107. doi: 10.1016/j.watres.2013.09.021.

Woodford, N., Fagan, E.J., and Ellington, M.J. (2006). Multiplex PCR for rapid detection of genes encoding CTX-M extended-spectrum (beta)-lactamases. *J Antimicrob Chemother* 57**,** 154-155. doi: 10.1093/jac/dki412.
